# Hematological inflammatory biomarkers as predictors of the severity and outcomes of bullous pemphigoid: A retrospective cohort study

**DOI:** 10.1016/j.jdin.2025.04.019

**Published:** 2025-09-10

**Authors:** Hsin-Hui Chen, Po-Wei Huang, Yung-Tsu Cho, Chia-Yu Chu

**Affiliations:** a Department of Dermatology, National Taiwan University Hospital and National Taiwan University College of Medicine, Taipei, Taiwan; b Graduate Institute of Clinical Medicine, College of Medicine, National Taiwan University, Taipei, Taiwan

**Keywords:** bullous pemphigoid, hematological inflammatory biomarkers

*To the Editor:* Bullous pemphigoid (BP), the most prevalent autoimmune bullous disease, predominantly affects the elderly. Its pathogenesis involves a complex interaction between autoantibodies targeting the hemidesmosomal proteins BP180 and BP230, neutrophils, eosinophils, and various cytokines.^1^ Systemic glucocorticoids remain the mainstay treatment, but prolonged immunosuppressive therapy increases morbidity and mortality in elderly patients, emphasizing the need to balance disease control with treatment-related complications.^2^

Identifying predictive biomarkers of BP severity is essential for clinical decision-making. Previous studies have identified associations between hematological inflammatory biomarkers, such as neutrophil-to-lymphocyte ratio (NLR), platelet-to-lymphocyte ratio (PLR), eosinophil counts, and disease severity in BP.^3^ However, the impact of these biomarkers on mortality and treatment response requires further research to clarify their prognostic and therapeutic significance. This study aimed to elucidate the associations between these biomarkers, disease severity, and clinical outcomes in BP.

We conducted a single-center retrospective cohort study of patients diagnosed with BP between July 2009 and December 2023. After applying strict diagnostic criteria and excluding confounding conditions, 144 of the initial 195 patients were enrolled. Disease severity was classified using the Bullous Pemphigoid Disease Area Index (BPDAI) into nonsevere (BPDAI <57, *n* = 97) and severe (BPDAI ≥57, *n* = 47) groups.

Baseline characteristics, including age, gender, and comorbidities, were similar between the nonsevere and severe BP groups. However, at initial diagnosis, the severe BP group showed significantly higher NLR (*P* = .028), PLR (*P* = .007), eosinophil-to-lymphocyte ratio (ELR, *P* = .016), neutrophil count (*P* < .001), eosinophil count (*P* = .018), and lower lymphocyte-to-monocyte ratio (LMR, *P* = .006) (Table I).

**Table I:** Baseline characteristics of patients with BP, stratified by disease severity

	Nonsevere BP (*n* = 97)	Severe BP (*n* = 47)	*P* value
Social demographics			
Age of diagnosis, mean (SD), y	75.99 (14.03)	71.79 (13.88)	.093
Gender, *n* (%)			.657
Male	54 (55.67)	28 (59.57)	
Female	43 (44.33)	19 (40.43)	
Diagnosis year, *n* (%)			.945
2009-2017	46 (47.42)	22 (46.81)	
2018-2023	51 (52.58)	25 (53.19)	
Comorbidities			
Hypertension, *n* (%)	57 (58.76)	24 (51.06)	.383
Diabetes mellitus, *n* (%)	50 (51.55)	27 (57.45)	.506
Dyslipidemia, *n* (%)	16 (16.49)	13 (27.66)	.117
Heart disease, *n* (%)	44 (45.36)	19 (40.43)	.576
Chronic kidney disease, *n* (%)	20 (20.62)	9 (19.15)	.837
Neurological disease, *n* (%)	45 (46.39)	18 (38.30)	.359
Disease severity and outcome			
BPDAI, mean (SD)			
Total score	31.55 (14.31)	80.28 (22.92)	**<.001**∗
Erosions/Blisters	17.97 (10.76)	48.11 (19.85)	**<.001**∗
Urticaria/Erythema	12.93 (7.89)	31.55 (17.02)	**<.001**∗
Mucosal activity	0.59 (2.33)	0.62 (1.11)	.924
Cumulative overall mortality			
1 y, *n* (%)	7 (7.22)	3 (6.38)	.961
3 y, *n* (%)	15 (15.46)	4 (8.51)	.458
5 y, *n* (%)	19 (19.59)	5 (10.64)	.207
Hematological inflammatory markers at initial diagnosis			
NLR, mean (SD)	5.22 (4.99)	7.68 (6.68)	**.028**†
PLR, mean (SD)	187.79 (128.06)	278.49 (202.09)	**.007**‡
PNR, mean (SD)	45.04 (22.34)	42.72 (19.29)	.544
LMR, mean (SD)	3.48 (1.81)	2.62 (1.45)	**.006**‡
RLR, mean (SD)	0.01 (0.01)	0.01 (0.01)	.246
ELR, mean (SD)	0.32 (0.44)	0.56 (0.58)	**.016**†
Neutrophil count, mean (SD), k/uL	6.36 (2.87)	8.34 (3.22)	**<.001**∗
Eosinophil count, mean (SD), k/uL	0.50 (0.74)	0.84 (0.86)	**.018**†
Hb, mean (SD), g/dL	12.32 (2.21)	11.98 (1.98)	.376

Values are presented as mean (SD) or number (percentage), as appropriate. Comparisons between patients with nonsevere and severe BP were conducted using Pearson’s chi-squared test or Fisher’s exact test for categorical variables and Student *t* test or Mann-Whitney *U* test for continuous variables, as appropriate.

Significant differences were observed in BPDAI scores and several hematological inflammatory markers.

Bolded *P* values indicate statistical significance (*P* < .05).

*BP*, Bullous pemphigoid; *BPDAI*, Bullous Pemphigoid Disease Area Index; *ELR*, eosinophil-to-lymphocyte ratio; *Hb*, hemoglobin; *LMR*, lymphocyte-to-monocyte ratio; *NLR*, neutrophil-to-lymphocyte ratio; *PLR*, platelet-to-lymphocyte ratio; *PNR*, platelet-to-neutrophil ratio; *RLR*, red blood cell distribution width-to-lymphocyte ratio.

∗ *P* < .001.

† *P* < .05.

‡ *P* < .01.

Univariable logistic regression identified elevated NLR, PLR, ELR, neutrophil count, and eosinophil count, as well as decreased LMR, as significant predictors of severe BP. To avoid multicollinearity, 2 separate multivariable models were constructed: model 1 (including NLR and ELR) showed ELR as an independent predictor (odds ratio [OR] = 2.499, *P* = .025), and model 2 (including absolute neutrophil and eosinophil counts) identified eosinophil count (OR = 1.996, *P* = .006) and PLR (OR = 1.004, *P* = .022) as independent predictors of severe BP (Table II). Kaplan-Meier analysis further demonstrated significantly higher 1-year mortality among patients with anemia (*P* = .045) or high ELR (>0.33, *P* = .024) (Supplementary Material, available via Mendeley at https://data.mendeley.com/datasets/nvyrvfcw6x/1).

**Table II:** Univariable and multivariable logistic regression analyses identifying predictors of severe BP

Variable	Univariable		Multivariable (model 1)		Multivariable (model 2)	
	Odds ratio (95% CI)	*P* value	Odds ratio (95% CI)	*P* value	Odds ratio (95% CI)	*P* value
Gender	1.174 (0.579-2.380)	.657				
Age	0.979 (0.955-1.004)	.096				
NLR	1.076 (1.011-1.146)	**.022**∗	0.981 (0.878-1.098)	.742		
PLR	1.004 (1.001-1.006)	**.004**†	1.004 (1.000-1.008)	.075	1.004 (1.001-1.007)	**.022**∗
PNR	0.995 (0.978-1.012)	.542				
LMR	0.720 (0.567-0.916)	**.007**†	0.899 (0.666-1.213)	.485	0.954 (0.712-1.278)	.753
RLR	1.156E+13 (0-1.583E+33)	.204				
ELR	2.499 (1.219-5.124)	**.012**∗	2.499 (1.120-5.578)	**.025**∗		
Neutrophil count (k/uL)	1.230 (1.090-1.388)	**<.001**‡	-		1.144 (0.998-1.310)	.053
Eosinophil count (k/uL)	1.686 (1.065-2.625)	**.025**∗	-		1.996 (1.219-3.270)	**.006**†
Hb (g/dL)	0.908 (0.789-1.093)	.374				

Variables significantly associated with severe BP in univariable logistic regression (*P* < .05) were included in multivariable models. The NLR and ELR are intrinsically correlated with neutrophil and eosinophil counts, respectively, as the ratios are derived from the same parameters. To prevent multicollinearity and ensure model stability, only 1 variable from each correlated pair was included in the multivariable analysis.

Two multivariable models were constructed: model 1 included inflammatory ratios (NLR, PLR, LMR, and ELR), while model 2 incorporated absolute cell counts (neutrophil and eosinophil) along with PLR and LMR, replacing the corresponding ratios.

The diagnostic performance of each model was evaluated using receiver operating characteristic curve analysis.

Model 1 (ELR-based model): area under the receiver operating characteristic curve = 0.722, optimal cutoff = 0.250, sensitivity = 0.87, specificity = 0.51, Youden index = 0.380.

Model 2 (eosinophil-based model): area under the receiver operating characteristic curve = 0.778, optimal cutoff = 0.215, sensitivity = 0.91, specificity = 0.56, Youden index = 0.476.

The corresponding logistic regression formulas are provided below:

Model 1: Logit(P) = −1.5444 − 0.0188 × NLR + 0.0038 × PLR − 0.1070 × LMR + 0.9159 × ELR.

Model 2: Logit(P) = −2.8235 + 0.0035 × PLR − 0.047 × LMR + 0.1341 × Neutrophil + 0.6913 × Eosinophil.

Bolded *P* values indicate statistical significance (*P* < .05).

*BP*, Bullous pemphigoid; *ELR*, eosinophil-to-lymphocyte ratio; *Hb*, hemoglobin; *LMR*, lymphocyte-to-monocyte ratio; *NLR*, neutrophil-to-lymphocyte ratio; *PLR*, platelet-to-lymphocyte ratio; *PNR*, platelet-to-neutrophil ratio; *RLR*, red blood cell distribution width-to-lymphocyte ratio.

∗ *P* < .05.

† *P* < .01.

‡ *P* < .001.

Eosinophils are involved in the inflammatory pathogenesis of BP, characterized by eosinophilic infiltration mediated by T-helper 2 cytokines and enhanced by thymic stromal lymphopoietin.^4^ The ELR provides additional prognostic value by reflecting the imbalance between eosinophils and lymphocytes, offering a more comprehensive measure of immune dysregulation. This complementary utility is further supported by Kaplan-Meier survival analysis, which demonstrated an association between elevated ELR and increased 1-year mortality, highlighting its potential relevance beyond the absolute eosinophil count. Our findings suggest that ELR may serve as a valuable biomarker for assessing disease severity and prognosis in BP, warranting further investigation. Additionally, our results corroborate previous evidence linking anemia to increased mortality, supporting a potential association between systemic inflammation and adverse outcomes.^5^

In conclusion, elevated ELR was identified as an independent predictor of severe BP, while anemia and high ELR were associated with increased mortality. These findings suggest a potential role for hematological inflammatory biomarkers in enhancing clinical assessment and guiding patient care in BP.

## Conflicts of interest

None disclosed.
